# AR and PI3K/AKT in Prostate Cancer: A Tale of Two Interconnected Pathways

**DOI:** 10.3390/ijms24032046

**Published:** 2023-01-20

**Authors:** Elisabetta Tortorella, Sabrina Giantulli, Alessandro Sciarra, Ida Silvestri

**Affiliations:** 1 Department of Molecular Medicine, Sapienza University of Rome, Viale Regina Elena 324, 00161 Rome, Italy; 2 Department of Urology, Sapienza University of Rome, Viale del Policlinico, 00161 Rome, Italy

**Keywords:** prostate cancer, androgen receptor signaling, PI3K/AKT/mTOR, PTEN, combination therapy

## Abstract

Prostate cancer (PCa) is the most common cancer in men. The androgen receptor (AR) has a pivotal role in the pathogenesis and progression of PCa. Many therapies targeting AR signaling have been developed over the years. AR signaling inhibitors (ARSIs), including androgen synthesis inhibitors and AR antagonists, have proven to be effective in castration-sensitive PCa (CSPC) and improve survival, but men with castration-resistant PCa (CRPC) continue to have a poor prognosis. Despite a good initial response, drug resistance develops in almost all patients with metastatic CRPC, and ARSIs are no longer effective. Several mechanisms confer resistance to ARSI and include AR mutations but also hyperactivation of other pathways, such as PI3K/AKT/mTOR. This pathway controls key cellular processes, including proliferation and tumor progression, and it is the most frequently deregulated pathway in human cancers. A significant interaction between AR and the PI3K/AKT/mTOR signaling pathway has been shown in PCa. This review centers on the current scene of different AR and PI3K signaling pathway inhibitors, either as monotherapy or in combination treatments in PCa, and the treatment outcomes involved in both preclinical and clinical trials. A PubMed-based literature search was conducted up to November 2022. The most relevant and recent articles were selected to provide essential information and current evidence on the crosstalk between AR and the PI3K signaling pathways. The ClinicalTrials.gov registry was used to report information about clinical studies and their results using the Advanced research tool, filtering for disease and target.

## 1. Introduction

Prostate cancer (PCa) is the most common cancer in men and the second-leading cause of cancer [1].

A more widespread prevention through screening with the prostate-specific antigen (PSA) has shown an increase in PCa incidence rate [2]. Most cases are diagnosed in the early stages (78% in localized stages and 12% with regional involvement), but 5% of cases are identified as already metastatic [3].

The time of diagnosis influences the prognosis. PCa can be a serious disease, but the majority of diagnosed patients do not die from it unless it becomes more aggressive and castration-resistant. Patients with localized PCa have a survival rate of 90% versus only 67% in metastatic PCa [4,5].

Treatment for localized PCa includes surgery (radical prostatectomy (RP)), radiotherapy (RT), or active surveillance, depending on the stage of the disease and other factors [3]. Despite the high rates of progression-free survival (PFS), about 20 to 30% of patients with PCa can experience cancer recurrence [6,7], characterized by rising serum PSA [4]. Clinical relapse occurs, on average, after 5 years (1–8 years) [8,9]. These patients with relapsed disease are either treated with salvage radiation therapy (SRT) or androgen deprivation therapy (ADT) [10]. ADT involves biochemical inhibition of male sex hormones, blocking the production of androgens in the testes via the hypothalamus–pituitary–gonadal (HPG) axis with luteinizing hormone-releasing hormone (LHRH) agonists (e.g., leuprolide) or antagonists (e.g., degarelix) [11,12,13].

Despite a good initial response, androgen-independent and castration-resistant prostate cancer (CRPC) can occur and the outcome is poor [14,15,16]. Both patients with CRPC and with metastatic disease at diagnosis can have improvements in overall survival (OS) with many treatments that include chemotherapy, such as docetaxel and, recently, cabazitaxel; radium-223, used for the treatment of bone; and denosumab, an antagonist of receptor activator of nuclear factor kappa-B ligand (RANKL). There is also increased interest in new androgen synthesis inhibitors, such as abiraterone acetate, or second-generation competitive AR antagonists, enzalutamide, as well as apalutamide and darolutamide [17,18,19,20,21,22,23].

Furthermore, clinical studies on agents that target the immune checkpoint, such as cytotoxic T-lymphocyte-associated protein 4 (CTLA4), programmed cell death protein 1 (PD1), or programmed death-ligand 1 (PD-L1), have been evaluated in the clinic [24]. To date, the Food and Drug Administration (FDA) has approved Sipuleucel-T immunotherapy, which is the only dendritic cell vaccine for asymptomatic or minimally symptomatic CRPC without visceral metastases [25]. In recent years, genetic DNA repair alterations were also found in some PCa patients and, in these cases, poly ADP ribose polymerase (PARP) inhibitors, including olaparib and rucaparib, both approved by the FDA, or talazoparib and niraparib have shown efficacy [26].

Hence, in this complex scenario focused on identifying both disease biomarkers and novel and effective treatments that will benefit patients with PCa, this review centers on the current scene of different Phosphatidylinositol-3-kinase (PI3K) signaling pathway inhibitors, either as monotherapy or in combination treatments in PCa, and the treatment outcomes involved in both preclinical and clinical trials.

## 2. Androgen Receptor Signaling Pathway

### 2.1. Androgens and Androgen Receptor

AR signaling has a pivotal role in prostate development and homeostasis as well as in prostate tumorigenesis [27,28], due to the fact that inhibition of AR signaling remains the mainstay therapeutic target in PCa [28].

Briefly, androgens, such as testosterone, are synthesized primarily by the Leydig cells in the testes and are tightly regulated by the hypothalamic–pituitary–gonadal (HPG) axis.

Once produced, testosterone mostly circulates bound to serum sex hormone-binding globulin (SHBG) and albumin [29]. Only the free form enters prostate cells. Intracellularly, testosterone is converted into a more potent 5α-reduced metabolite of testosterone, 5α-dihydrotestosterone (DHT), essential for the growth and survival of these cells. Circulating testosterone and DHT represent the main native agonists for AR.

AR is a member of the steroid and nuclear hormone receptor superfamily and is encoded by the AR gene mapped on the long arm of the X-chromosome (locus: Xq11-q12) [30,31]. The AR consists of four domains: an amino-terminal transcriptional domain (NTD), DNA-binding domain (DBD), a hinge region, and a carboxy-terminal ligand-binding domain (LBD) [32].

In the cytoplasm, AR is bound with heat-shock proteins (HSPs)-90, HSP-70, HSP-56, and other chaperone proteins to protect the receptor against degradation [32,33].

The binding of the ligand promotes the dissociation of AR from these complexes and AR can shuttle to the nucleus, where it dimerizes and binds to the androgen response elements (AREs) [33,34]. AREs also include genes that are involved in PSA expression, androgen biosynthesis, DNA synthesis and repair, cell cycle regulation, and proliferation [35,36].

Furthermore, AR transactivation is modulated by coregulators. At least 300 proteins have been identified that can coactivate or co-repress AR-dependent transcription [37].

In addition to the DNA-binding-dependent actions of the AR, commonly referred to as genomic or canonical, the complex androgen-AR can also trigger a signal through a non-DNA-binding-dependent mechanism, referred to as nongenomic. The non-canonical androgen signaling pathways can be classified as non-genomic pathways (triggered by androgens) and outlaw pathways (androgen-independent, but AR-dependent) [38].

Non-genomic androgen signaling pathways include androgen-induced activation of G-protein-coupled receptors (GPCRs), the protein kinase A (PKA) pathway, and via AR variants [39].

### 2.2. Therapies Targeted to AR Signaling

Considering that androgens and AR signals are essential regulators in the growth of the normal prostate and play a key role in PCa pathogenesis, they represent a target for many agents, used both as monotherapy or in combination, with the intent to treat and improve the outcome of distinct clinical phenotypes of prostate disease: localized, CSPC, mCSPC, CRPC, and mCRPC. Therapies that aim to inhibit AR signaling, collectively called AR signaling inhibitors (ARSIs), have undergone a significant advance since the 1940s. At that time, Huggins and Hodges demonstrated the therapeutic effect of gonadal testosterone deprivation via orchiectomy [40]. Nowadays, the available drugs target the gonadotropin-releasing hormone (GnRH) to prevent luteinizing hormone (LH) releasing, cytochrome P450 17α-hydroxylase/17,20-lyase (CYP17A1) to restrain androgen synthesis, or directly AR, to inhibit AR transcriptional activity [16,17,18,19,20].

#### 2.2.1. Androgen Deprivation Therapy (ADT)

ADT via surgical or chemical castration has become crucial in the treatment of PCa and metastatic disease with the aim to suppress serum testosterone to castration levels and to block the activation of the AR [14,15]. Gonadotropin-releasing hormone (GnRH) agonists, such as histrelin, goserelin, leuprolide, and triptorelin, approved in the 1980s by the FDA, continue to be the mainstay of ADT. Their action is based on the activation of GnRH receptors, leading to an increase in follicle-stimulating hormone (FSH), LH, and, consequentially, testosterone production. Despite this, the continuous stimulation, which is the opposite of the physiological pulsatile action of GnRH receptors, leads to their downregulation and subsequent desensitization of the pituitary gland to the effects of GnRH [41]. Hence, testosterone production decreases to castration levels over 3 to 4 weeks [42]. These agents may be associated with an increased likelihood of adverse events compared to orchiectomy, including cardiovascular events, fractures, and peripheral arterial disease [43,44]. GnRH antagonists induce testosterone suppression by directly inhibiting GnRH receptors. Injectable degarelix [45] and relugolix [46], oral GnRH antagonists, were approved by the FDA in 2009 and 2020, respectively, for advanced PCa. It seems that the risk of cardiovascular events in men taking GnRH antagonists is lower compared to those taking GnRH agonists [47]. The androgen synthesis inhibitor targets CYP17, a member of the cytochrome P450 family. CYP17 catalyzes and converts precursor steroids to testosterone, DHT, and dehydroepiandrosterone (DHEA). Abiraterone acetate is a selective inhibitor of 17α-hydroxylase/C17,20-lyase (CYP17) and blocks the production of androgen in the adrenal glands, testes, placenta, as well as in PCa cells [48]. Because of interaction with numerous targets, steroid supplementation is necessary to overcome cortisol insufficiency and limit some adverse effects (AEs) [20]. Some studies have demonstrated that a dose of 800 mg once daily is effective in suppressing testosterone to below detectable levels in humans and provided preliminary insight into its safety profile [49]. This drug was FDA-approved in 2011 for patients with mCRPC who had received prior chemotherapy associated with prednisone, and in 2012, for patients with mCRPC and, together with ADT, for metastatic high-risk CSPC [49,50,51]. Furthermore, in the early 1980s, it was shown that castration levels of serum testosterone can be induced also by high doses of the imidazole antifungal agent ketoconazole (Nizoral), by blocking cytochrome P450 enzymes (including CYP17A1) [52].

#### 2.2.2. Androgen Receptor Antagonists

Androgen receptor antagonists (ARAs) bind to the LBD of the AR and prevent the binding of testosterone and DHT, so that AR translocation into the nucleus is inhibited and, consequently, the interactions with AREs. The non-steroidal antiandrogens flutamide (Eulexin), nilutamide (Anandron) and bicalutamide (Casodex) represent that the first generation of ARAs do not completely block AR activity [53] and show a short-lived benefit [16]. Enzalutamide (MDV3100), a second-generation ARA, has higher affinity for the AR and minimal no-agonist activity [54]. After the encouraging results from several clinical trials [21,55], the FDA approved enzalutamide in the pre- and post-chemotherapy mCRPC (2012), CRPC (2018) as well as in mCSPC (2019). Apalutamide (ARN 509) has a similar mechanism of action as enzalutamide [56] but a greater efficacy [23]; the FDA approved apalutamide for the treatment of patients with non-metastatic (nm) CRPC (2018) and for patients with mCSPC (2019). Apalutamide significantly improves OS and radiographic progression-free survival (rPFS) in patients with mCSPC receiving ongoing ADT, as analyzed in TITAN [57]. Darolutamide (ODM-201) has a particular molecular structure that is distinct from other AR antagonists [58,59]. Darolutamide and its metabolite have a tighter binding to the AR than enzalutamide and apalutamide with lower toxicity and higher OS. Moreover, Darolutamide can also antagonize some mutations within AR, which confer resistance for enzalutamide and apalutamide. It is the last AR inhibitor approved by the FDA for treatment of nmCRPC (2019) and for mCSPC (2022) [55,56].

### 2.3. Alterations in AR Signaling

Despite these therapies suppressing most PCas, some high-risk prostate cancers gradually progress to CRPC and mCRPC. The AR signaling pathway plays a central role also in the progression of PCa during hormonal therapy [60,61,62]. Several mechanisms can restore AR signaling, including intracrine androgen synthesis, AR overexpression and amplification, point mutations, acquisition of constitutively active AR splice variants, deregulated AR coactivators/corepressors that sensitize AR in response to ligand binding, and, finally, ligand-independent signaling [3]. These alterations are not exclusive and can even coexist in the same patient [62].

#### 2.3.1. Amplification of the AR Gene and Overexpression of the AR Protein

AR gene amplification leads to an overexpression of the AR protein and is the most common genetic mutation among patients with CRPC [63]. Results from studies using fluorescence in situ hybridization (FISH) have shown that AR amplification is the most frequent genetic alteration detected in CRPC, observed in more than 50% of cases, while it is only rarely detected in untreated primary tumors, suggesting that AR amplification could be an adaptive response to ADT. AR gene amplification was also analyzed at the mRNA level. By using reverse-transcription polymerase chain reaction (RT-PCR), it has been observed that the expression of AR mRNA in CRPC with AR amplification was two-fold higher than CRPC without AR amplification. Elevated AR protein levels were also linked to CRPC [64].

Furthermore, it has been shown that AR gene amplification is frequently detected in the circulating tumor cells (CTCs) of patients with CRPC [65]. As well, AR amplification is more common in patients resistant to both enzalutamide and abiraterone (more enzalutamide than abiraterone) [66], suggesting that AR amplification could be a mechanism responsible for ADT resistance [34]. Finally, ADT may induce upregulation of AR transcription by disrupting the AR negative-feedback loop, then resulting in elevated AR-FL and AR-Vs protein levels [67,68]. Moreover, in CRPC, decreased androgen levels may relieve AR suppression of genes mediating DNA synthesis, thereby contributing to tumor cell proliferation [67].

#### 2.3.2. Point Mutations in Androgen Receptor

Point mutations in the AR gene are rarely detectable in early-stage PCa but are detected in 15–20% of CRPC patients and up to 40% of CRPC patients treated with AR antagonists, most frequently in the LBD, followed by the NTD [66,69]. The acquisition of AR mutations results in broadening ligand specificity by weak adrenal androgens and other steroid hormones, including DHEA, progesterone, estrogen, and glucocorticoids, as well as in reversing antagonists into agonists [70]. T878A was the first identified point mutation resulting in the loss of specificity for the agonist. Progesterone, estrogens, flutamide, bicalutamide, and enzalutamide can activate AR carrying the T878A point mutation [71]. Moreover, T878A also causes resistance to second-generation AR agonists [66]. Resistance to enzalutamide is also induced by the point mutation F876L, in the LBD domain [64,65,66]. Finally, H875Y, T878A, and T878S mutations detected in CRPC patients induce agonist effects by enzalutamide and apalutamide [70]. Mutations, such as T878A or L702H, have been identified in the plasma of 13% of CRPC patients progressing during abiraterone treatment. Other AR point mutations, T877A, L702H, and H875Y, have been detected in cell-free DNA from patients with CRPC and have also been associated with resistance to abiraterone and enzalutamide [72].

#### 2.3.3. AR Splice Variants

Androgen receptor splice variants (AR-Vs) are the main causes of abnormalities in AR regulation [73,74]. AR-Vs are derived by a splicing mechanism from full length and are truncated receptors with a modified LBD domain, but normal NTD and DBD domains, required for endogenous AR functions. The effect of the previously reported modification is that AR may still mediate signaling in the absence of the ligand, but PC cells become resistant to many ARSIs, including abiraterone and enzalutamide. Generally, these AR-Vs have been associated with poor prognosis in advanced PCa [75]. AR-V7 is the most prevalent variant among more than 20 AR-Vs identified in CRPC, followed by the exon-skipping AR-v567 [74,76]. AR-V7 has been detected in 75% of mCRPC but only rarely in early-stage disease (<1%) [77]. This variant has also been associated with an increased risk of recurrent disease after prostatectomy in CSCR and with low survival in CRPC. Furthermore, the identification of AR-V7 mRNA levels in whole blood of mCRPC patients receiving abiraterone has been correlated with poor outcome [78]. This and other studies support that AR-V7 detection in patients with CRPC could be correlated with resistance to enzalutamide and abiraterone [79]. Some studies are aimed at better understanding if AR-V7 protein expression is simply associated with enhanced AR-FL expression as a marker of resistance to ARSI or whether a critical level of AR-V7 is required for such ARSI refractory lethal cancer growth [80,81,82]. Some studies have focused on understanding the functional differences, as compared to canonical AR-FL. AR-V7 exhibits fast nuclear import kinetics via a pathway distinct from the nuclear localization signal-dependent importin-α/β pathway used by AR-FL. The dimerization box domain, known to mediate AR dimerization and transactivation, is required for AR-V7 nuclear import but not for AR-FL. In the nucleus, AR-V7 is transcriptionally active and exhibits unusually high intranuclear mobility and transient chromatin interactions, unlike the stable chromatin association of liganded AR-FL. The high intranuclear mobility of AR-V7 together with its high transcriptional output suggest a hit-and-run mode of transcription [83,84]. These mechanisms regulating AR-V7 activity could offer the opportunity to develop selective therapeutic interventions [85].

AR-V567es is one of the two most frequently observed AR variants. It has exons 5–7 spliced out and only contains a small portion of the LBD. Results from studies using transgenic mice have shown that ARV567es induces carcinogenesis and accelerates tumor progression [86]. Studies are ongoing to explore if AR-V567es confers relatively greater taxane sensitivity than AR-V7 [87,88].

## 3. PI3K/AKT/mTOR Signaling Pathway

The PI3K/AKT/mammalian target of rapamycin (mTOR) pathway is considered to be a pivotal intracellular signaling pathway and its hyperactivity is correlated with tumor progression in a wide assortment of cancers, counting breast, gastric, ovarian, colorectal, prostate, glioblastoma, and endometrial cancers [89]. PI3K kinase activation constitutes a central mechanism between upstream growth signals and downstream signal transduction mechanisms involved in numerous cellular processes, such as protein synthesis, metabolism, inflammation, cell survival, motility, and tumor progression. PI3K is a large family of lipid enzymes capable of phosphorylating the 3′-OH group of phosphatidylinositol present on the plasma membrane. It was discovered more than 25 years ago and initially associated with the transforming ability of viral oncoproteins. Three classes of PI3Ks (class I, class II, and class III) have been identified in mammals. Kinases belonging to class IA consist of a catalytic subunit and a regulatory subunit. The catalytic subunits p110α, p110β, or p110δ are encoded by the PIK3CA, PIK3CB, and PIK3CD genes, respectively. In contrast, the regulatory subunits consist of p85α (in the isoforms p85α, p55α, p50α), p85β, and p55γ, which are encoded by the *PIK3R1*, *PIK3R2*, and *PIK3R3* genes. Class IB consists of only one catalytic subunit, P110γ, and two regulatory subunits, p84 and p101 [90]. Class II includes three different monomeric isoforms and remains the most enigmatic of all PI3Ks, although recent studies have provided new clues about its role in signal transduction [91]. Finally, the only member of class III is known as Vps34 (Vacuolar protein signal 34), expressed in all eukaryotic organisms. Vps34 was first discovered in yeast and is implicated in the integration of cellular responses and changes in nutritional status [92]. A variety of signals stimulate PI3K activity, mainly through receptor tyrosine kinases (RTKs), but also through GPCRs and oncogenes, such as *Ras*, that directly bind p110. After stimulation, the catalytic subunit of PI3K phosphorylates phosphatidylinositol-4,5-bisphosphate (PIP2) to phosphatidylinositol-3,4,5-triphosphate (PIP3), which acts as a second messenger to recruit a series of proteins containing homology domain with the pleckstrin (PH) of the cell membrane. Uncontrolled signaling of PI3K is very common in cancer, also due to the different roles played by its catalytic subunits p110α and p110β. Mutations in the *PI3KA* gene encoding p110α have been shown in cancer cells from the colon, lung, prostate, liver, and brain [93]. This gene, in addition to being involved in the processes of cell cycle regulation and growth, acquires a very important role in endothelial cells, promoting angiogenesis and, thus, the formation of a vascular network essential for the delivery of nutrients and oxygen, which can ultimately ensure a pathway of metastasis from the primary lesion. In oncogenesis, the p110α isoform is required for tumors driven by activated receptor tyrosine kinases and oncogenes. The p110β is mainly required for GPCR downstream signaling but it was also found to be essential for the development of high-grade prostatic intraepithelial neoplasia (HG-PIN) [94]. In an animal model of Phosphatase and Tensin homolog (*PTEN*)-deficient PCa, ablation of p110β, but not that of p110α, impeded tumorigenesis, with a concomitant diminution in AKT phosphorylation [95]. Consistently, data from the latest studies suggested that while blockade of p110α had negligible effects in the development of *PTEN*-null CRPC, genetic or pharmacological disruption of p110β dramatically slowed the initiation and progression of CRPC [94,95,96].

A key molecule in the regulation of PI3K/AKT is *PTEN*. The function of *PTEN* as an oncosuppressor is carried out through its phosphatase activity; it dephosphorylates PIP3 to PIP2, negatively regulating the activation of the PI3K/AKT pathway. This phosphatase can act on both lipids and proteins, and acts by inhibiting cell proliferation and inducing apoptosis. Mutations to *PTEN* inhibit its oncosuppressive activity. Two major mutations affect the phosphatase domain: one results in the loss of phosphatase activity on both lipids and proteins while the other impairs phosphatase activity on protein substrates. In addition to regulating the PI3K/AKT signaling pathway, *PTEN* has many other critical roles in tumors, including genomic instability, tumor cell renewal, cell senescence, cell migration, and metastasis. Finally, *PTEN* plays a significant role in regulating the tumor microenvironment [97]. Mutations in the *PTEN* gene have been observed in breast, prostate, endometrial, ovarian, colon, melanoma, glioblastoma, and lymphoma cancer [98]. Studies in animal models have also shown that the loss of a single copy of the *PTEN* gene is sufficient to disrupt cell signaling and initiate uncontrolled cell growth [99]. PI3K activation leads to phosphorylation and then activation of AKT, or protein kinase B (PKB), a serine/threonine kinase of the AGC family of kinases. It exists in three structurally similar isoforms: AKT1, AKT2, and AKT. The three isoforms are composed of characteristic domains. The Pleckstrin Homology (PH) domain has a remarkably conserved tertiary structure, although the amino acid sequence may differ; this domain is responsible for binding to PIP3. The LIN domain, of 39 amino acids is the hinge region connecting the PH domain with the catalytic domain, which is poorly conserved among AKT isoforms (17–46% identical) and has no significant homology with any other human protein. The kinase domain extends from amino acids 148–411 and terminates in a hydrophobic regulatory motif (CTD), ATP-binding portion of the enzyme; the ATP-binding site of 25 residues has 96–100% homology across the three isoforms. The C-terminal hydrophobic domain appears to be conserved in the AGC family of kinases. These hydrophobic residues play a critical role in the complete activation of AKT for substrate phosphorylation. Within it is another key residue for enzyme activation, Ser473. While AKT1 is ubiquitously expressed at high levels, except for the kidney, liver, and spleen, AKT2 expression is high in insulin-sensitive tissues, such as brown fat, skeletal muscle, and liver. AKT3 expression is ubiquitous, although low levels of expression have been found in liver and skeletal muscle. These different isoforms appear to be implicated in specific functions. For example, amplification and overexpression of AKT2 correlate with increased cell motility and invasion, whereas increased AKT3 activity appears to contribute to the aggressiveness of steroid-hormone-insensitive tumors [100]. All three isoforms are activated through phosphorylation: the first occurs on a threonine residue while the second occurs on a serine residue in the hydrophobic motif. Once activated, AKT recognizes and phosphorylates serine or threonine residues of numerous substrates, such as tuberosis sclerosis complex 2 (TSC 2), glycogen synthase kinase 3 (GSK 3), forkhead box transcription factors (FOXO), p21WAF1/CIP1, p27KIP1, caspase-9, Bcl-2 associated death promoter (BAD), and inducible Nitric Oxide Synthase (iNOS), which regulate numerous processes that coordinate cell life and death, metabolism, and angiogenesis. Hyperactivation of AKT has been shown in numerous cancers, such as multiple myeloma, lung cancer, glioblastoma, breast cancer, prostate cancer, etc. [101]. The best-studied downstream substrate of AKT is mTOR kinase. AKT can directly phosphorylate and activate mTOR and can cause indirect activation of mTOR by phosphorylating and inactivating tuberous sclerosis 2, also called tuberin (TSC2), which normally inhibits mTOR. The consequence of mTOR activation is increased protein translation [102]. Finally, it has recently been shown that AKT activity can be negatively regulated by the PH domain of leucine repeat sequence-rich phosphatase (PHLPP), which specifically dephosphorylates the hydrophobic motif of AKT (Ser473 in Akt1) [103]. mTOR (mammalian target of rapamycin) is a serine/threonine kinase that regulates cell growth, proliferation, motility and survival, transcription, and protein synthesis. mTOR plays an important role in regulating the body’s energy balance and weight; it is activated by amino acids, glucose, insulin, and other hormones involved in regulating metabolism. Recent studies have shown that mTOR is not only a substrate of AKT but also a crucial activator of AKT. In fact, mTOR forms a complex (TORC2) with the protein Rapamycin-insensitive companion of mTOR (RICTOR) and then directly phosphorylates the Ser473 of AKT [104]. Activation of TORC2 could then explain the sequestration of newly formed mTOR molecules within cells during long-term rapamycin treatments. In fact, this drug is particularly effective in inducing apoptosis and suppressing the proliferation of AKT-overexpressing cells because, over time, it interferes with the reassembly of the complex by joining it [105].

## 4. Crosstalk between AR and PI3K Signaling

**Figure 1:**
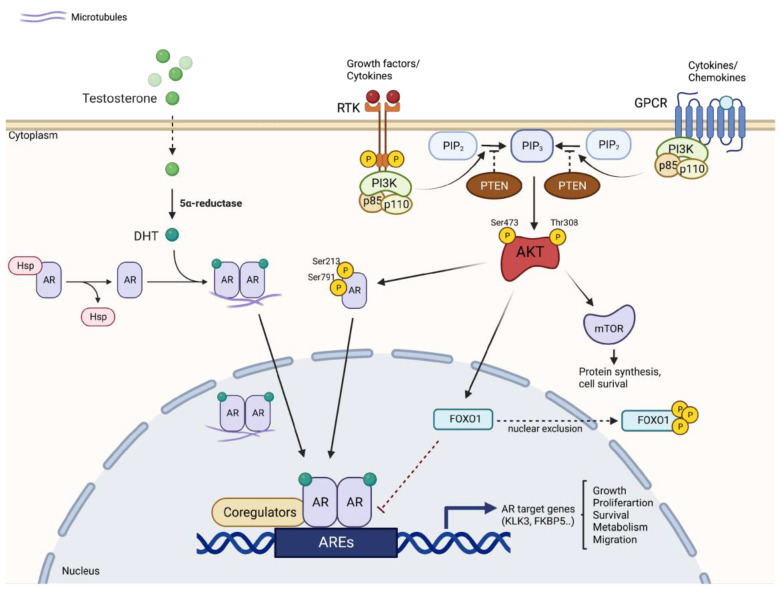
Crosstalk between PI3K/AKT/mTOR and AR signaling pathways. The two signaling pathways are closely connected and regulated according to a reciprocal feedback mechanism. AR inhibition reduces FKBP5 levels, inhibiting PHLPP-mediated suppression of AKT, thereby activating AKT. Activation of AKT then generates upregulation of AR by several mechanisms, including direct phosphorylation of AR and nuclear exclusion of FOXO1. AKT: protein kinase B; AR: androgen receptor; ARE: androgen response element; FKBP5: FK506 binding protein 5; FOXO1: forkhead box transcription factor 1; GPCR: G-protein coupled receptor; mTOR: mammalian target of rapamycin; PIP3: phosphatidylinositol-3,4,5-triphosphate; PHLPP: PH domain of leucine repeat sequence-rich phosphatase; PSA: prostate-specific antigen; PTEN: phosphatase and tensin homolog; RTK: receptor tyrosine kinase.

The PI3K/AKT/mTOR signaling pathway has been shown to be deregulated in a wide range of cancers. Genetic alterations have been identified in all components of this signaling pathway. In PCa, the PI3K/AKT/mTOR pathway is deregulated in 42% of localized and 100% of advanced disease cases, indicating that alterations in these signals might be an essential prerequisite for the development of CRPC [106]. The existence of negative feedback regulation within AR and PI3K/AKT signaling networks has been demonstrated [107] (Figure 1). Thus, gene mutations and amplifications, and changes in mRNA expression in components of the PI3K pathway, are strictly correlated with the prognosis of PCa patients. For example, reduced expression of *PTEN* is associated with higher Gleason, biochemical recurrence after prostatectomy, and shorter time to metastatic progression [108]. In addition, high levels of phospho-4EBP1 and eI4E are associated with increased mortality in patients with PCa, indicating that effectors further down the pathway are also predictive of disease progression [109].

Results from studies in knockout (KO) and transgenic mouse models have also clearly shown the role of PI3K/AKT/mTOR in the development of PCa. Specifically, overexpression of AKT or biallelic loss of the oncosuppressor *PTEN* in prostate epithelial cells leads to hyperactivation of the pathway and is sufficient for PCa development in vivo [110]. *PTEN* deletion has also been shown to inhibit the progression of PCa in mouse models with conditional KO of mTOR [111]. Others have also shown in vivo that the progression of PCa is reduced when *PTEN* and RICTOR, a subunit of mTORC2 complex, are lost [112]. This demonstrates that the progression of PCa can be sufficiently induced by the hyperactivation of PI3K/AKT/mTOR. Loss of the oncosuppressor *PTEN* and subsequent uncontrolled activation of the PI3K signaling pathway has been found in 40% of primary tumors and 70% of metastatic forms [113]. Moreover, AKT was shown to phosphorylate AR at Ser-210 and Ser-791, but the effect on AR activity and protein stability is debated, as studies have demonstrated either repression or activation of AR function [114]. From a functional point of view, AKT repressed AR transactivation in a reporter assay in AR-insensitive DU145 mPCa cells when the AR was exogenously expressed [115]. However, in the androgen-sensitive PCa LNCaP cell line, AKT phosphorylation activated the PSA reporter and promoted cell survival [115]. These differential effects suggest that AKT-mediated AR phosphorylation on AR function could be attributed to cell context. The AKT pathway is considerably sensitive to feedback regulation. Furthermore, inhibition of the PI3K pathway also stimulates the upstream of HER2/3, thereby activating the androgen receptor axis in murine and human tumors with PTEN deletion [116].

On the other hand, inactivation of AR would lead to over-regulation of the PI3K/AKT pathway, which has been correlated with altered control of cell growth and survival, increased metastatic competence, angiogenesis, and resistance to chemotherapy.

Indeed, it has been demonstrated that AR inhibition activates AKT signaling by reducing the expression of the AKT phosphatase PHLPP [117]. AR blockade reduces FKBP5 levels, then impairing PHLPP function and leading to upregulation of pAKT [118].

Thus, these two pathways are regulated by a reciprocal feedback mechanism in that the inhibition of one inactivates the other, allowing for cancer cell survival and progression.

### Combination Therapy

Because inhibiting either AR or AKT often activates the other, a combination therapy might be advantageous. Over 40 compounds targeting key components in the PI3K-induced signaling pathway have been investigated to date. AZD5363, an inhibitor of all isoforms of Akt, has been reported to inhibit proliferation and induce apoptosis in prostate cancer cell lines expressing AR and has antitumor activity in vivo in androgen-sensitive LNCaP xenograft models resistant to castration [119]. However, resistance occurs already after about 30 days of treatment. This is proposed to be since AZD5363 induces an increase in the binding affinity of AR to AREs and an increase in the transcriptional activity of AR and the expression of AR-dependent genes, such as PSA and NKX3.1. These effects were overcome by the combination of AZD5363 and the earlier antiandrogen Bicalutamide, resulting not only in a synergistic inhibition of cell proliferation and induction of apoptosis in vitro, but also in a prolongation of tumor growth inhibition and PSA stabilization [120]. Moreover, clinical data from the latest ongoing clinical trials support the hypothesis that combinatorial therapies may have a good response in treating advanced PCa (Table 1). The phase II ProCAID clinical trial suggested that addition of capivasertib (pan-AKT inhibitor) to docetaxel improved OS benefit in mCRPC patients. Median OS was 25.3 months for capivasertib plus docetaxel versus 20.3 months for placebo plus docetaxel (hazard ratio (HR) 0.70, 95% confidence interval (CI) 0.47–1.05; nominal *p* = 0.09) [121]. Another pan-AKT inhibitor, Ipatasertib, has been used in the recent randomized, double-blind, phase III trial combined with abiraterone (IPATential150). This combination led to prolonged radiographic progression-free survival and antitumor activity over a placebo with abiraterone among patients with mCRPC with PTEN loss (median 18.5 vs. 16.5 months, HR = 0.77; *p* = 0.0335) [122]. The phase I/II study investigating AZD8186, a potent and selective inhibitor of PI3K, supported combination treatment with abiraterone acetate [123]. Moreover, a phase II trial of everolimus (mTOR inhibitor) plus bicalutamide showed encouraging efficacy in men with bicalutamide-naïve CRPC [124]. It must be mentioned, however, that one of the limitations of the use of PI3K/AKT inhibitors is undoubtedly the occurrence of AEs, usually hyperglycemia, rash, and diarrhea. For this reason, numerous studies are focusing on understanding the mechanisms and management of toxicity. In addition, new phase I studies are aimed at optimizing the dosing schedule to improve drug-related toxicity. Noteworthily, most of the clinical trials to date are directed towards patients with advanced or mCRPC, which are very different from the earlier, localized, high-risk disease. Hence, the effects of the combined targeting of AR and PTEN/AKT pathways in the setting of localized prostate cancer need to be investigated.

## 5. Conclusions

The high incidence of prostate cancer in the global male population has resulted in many efforts being channeled into finding the best therapeutic strategies. In particular, the prognosis of mCRPC remains very poor to date since patients develop resistance to treatments. Indeed, there is currently no single therapeutic choice for these patients, but recommended therapies involve the use of agents targeting different signaling pathways, often in combination or as neoadjuvant therapies. Many studies have confirmed the role of the PI3K/AKT/mTOR signaling pathway in the development of treatment resistance and tumor progression. Indeed, activation of PI3K increases proliferation and prevents apoptosis of prostate cancer cells, while inactivation promotes cell cycle arrest in the G phase. On the other hand, there is evidence that AR inhibition promotes upregulation of the PI3K pathway, and vice versa, via a negative feedback mechanism.

Therefore, studies aiming to understand the key mechanisms that induce resistance within this continuous crosstalk between the two signaling pathways are needed to improve the outcome of patients with prostate cancer.

## Figures and Tables

**Table 1:** Clinical studies on PI3K/AKT/mTOR pathway inhibitors in PCa.

*Target*	*Agent*	*Phase*	*Regimen*	*Study Population*	*Status*	*Clinicaltrial.Gov ID*
AKT	MK2206	II	+ Bicalutamide	High-Risk of Progression PCa with rising PSA	Active, not recruiting	NCT01251861
		I	+ hydroxychloroquine	Stage IV PCa	Active, not recruiting	NCT01480154
	AZD5363 (Capivasertib)	I	+ Enzalutamide	Advanced Solid Tumors Harboring Mutations in AKT1, AKT2, or AKT3	Active, not recruiting	NCT03310541
		III	+ Docetaxel	mCRPC	Recruiting	NCT05348577
		II	+ Abiraterone acetate	High Risk Localized PCa With PTEN Loss	Not yet recruiting	NCT05593497
		I/II	+ Docetaxel/Prednisolone	mCRPC	Completed	NCT02121639
	Afuresertib	I/II	+ LAE001/prednisone	mCRPC	Recruiting	NCT04060394
PI3K	AZD8186	I	+ Docetaxel	mPCa with PTEN or PIK3CB mutations	Active, not recruiting	NCT03218826
		I	+ Abiraterone acetate	Advanced CRPC	Completed	NCT01884285
	GDC-0068 (Ipatasertib)	I/II	+ Atezolizumab	PTEN-loss CRPC	Recruiting	NCT03673787
		III	+ Abiraterone acetate and prednisone/prednisolone	mCRPC	Active, not recruiting	NCT03072238
		II	+ Abiraterone acetate and prednisone/prednisolone	CRPC previously treated with Docetaxel	Completed	NCT01485861
		I	+ Rucaparib	Advanced PCa	Completed	NCT03840200
	GSK2636771	I	+ Enzalutamide	PTEN(-) mCRPC	Completed	NCT02215096
	BKM120 (Buparlisib)	Ib	+ Abiraterone acetate	CRPC	Completed	NCT01634061
	LY3023414	II	+ Enzalutamide	mCRPC	Completed	NCT02407054
	CYH33	I	+ Olaparib	PCa with DDR and/or PIK3CA mutations	Recruiting	NCT04586335
mTOR	RAD001 (Everolimus)	III	Monotherapy	CRPC patients with PI3K-AKT-mTOR signaling pathway deficiency	Not yet recruiting	NCT03580239
		II	Monotherapy	mCRPC	Completed	NCT00636090
		I	+ Apalutamide	mCRPC after treatment with Abiraterone acetate	Completed	NCT02106507
		I	+ standard radiation therapy	PCa with rising PSA following RP	Completed	NCT01548807
		II	+ Carboplatin/Prednisone	mPCa that progressed after docetaxel	Completed	NCT01051570
		I/II	+ Docetaxel/Prednisone	mCRPC	Completed	NCT00459186
		II	Monotherapy	HRPC	Completed	NCT00629525
		I/II	+ Docetaxel/Bevacizumab	Advanced PCa	Completed	NCT00574769
	CCI-779 (Temsirolimus)	I/II	+ Cixutumumab	mPCa	Completed	NCT01026623
		II	+ conventional surgery	newly diagnosed PCa at high risk of relapse	Completed	NCT00071968
	CC-115	I	Monotherapy	PCa	Completed	NCT01353625
	MLN0128 (Sepanisertib)	II	Monotherapy	CRPC	Completed	NCT02091531
	AZD2014	I	Monotherapy prior to RP	High Risk PCa	Completed	NCT02064608

(+) indicates co-treatments; ADT: androgen deprivation therapy; CRPC: castration-resistant prostate cancer; HRPC: hormone-resistant prostate cancer; mPCa: metastatic prostate cancer; mCRPC: metastatic castration-resistant prostate cancer; PSA: prostate-specific antigen; RP: radical prostatectomy.

## Data Availability

No new data were created or analyzed in this study. Data sharing is not applicable to this article.

## Abbreviations

ADT	androgen deprivation therapy
AGC	protein kinase A, G and C families
AKT	protein kinase B
AR-FL	androgen receptor full length
AR-V	androgen receptor splice variants
AR	androgen receptor
ARA	androgen receptor antagonist
ARE	androgen response element
ARSI	androgen receptor signaling inhibitor
ATP	adenosine triphosphate
BAD	Bcl-2-associated death promoter
BAT	bipolar androgen therapy
CRPC	castration-resistant prostate cancer
CSPC	castration-sensitive prostate cancer
CTC	circulating tumor cells
CTD	hydrophobic regulatory motif domain
CTLA4	cytotoxic T-lymphocyte-associated protein 4
CYP17A1	cytochrome P450 17α-hydroxylase/17,20-lyase
DBD	DNA-binding domain
DHEA	dehydroepiandrosterone
DHT	5α-dihydrotestosterone
DNA	deoxyribonucleic acid
FDA	Food and Drug Administration
FISH	fluorescence in situ hybridization
FKBP5	FK506 binding protein 5
FOXO	forkhead box transcription factors
FSH	follicle-stimulating hormone
GnRH	gonadotropin-releasing hormone
GPCR	G-protein-coupled receptor
GSK 3	glycogen synthase kinase 3
HER	human epidermal growth factor receptor
HPG	hypothalamus–pituitary–gonadal axis
HSP	heat-shock protein
iNOS	inducible nitric oxide synthase
KO	knockout
LBD	ligand-binding domain
LH	luteinizing hormone
LHRH	luteinizing hormone-releasing hormone
LNCaP	androgen-sensitive human prostate adenocarcinoma cells
mCRPC	metastatic castration-resistant prostate cancer
mCSPC	metastatic castration-sensitive prostate cancer
mRNA	messenger ribonucleic acid
mTOR	mammalian target of rapamycin
mTORC1/2	mTOR complex ½
nmCRPC	non metastatic castration-resistant prostate cancer
NOS	nitric oxide synthase
NTD	amino-terminal transcriptional domain
OS	overall survival
p70S6K	p70S6 kinase
PARP	poly ADP-ribose polymerase
PCa	prostate cancer
PCR	polymerase chain reaction
PD-L1	programmed death-ligand 1
PD1	programmed cell death protein 1
PDK1	phosphoinositide-dependent kinase 1
PFS	progression-free survival
PH	pleckstrin homology domain
PHLPP	PH domain of leucine repeat sequence-rich phosphatase
PI(3,4)P2	phosphatidylinositol-3,4-biphosphate
PI(4,5)P2	phosphatidylinositol-4,5-biphosphate
PI3K	phosphatidylinositol-3-kinase
PIP3	phosphatidylinositol-3,4,5-triphosphate
PKA	protein kinase A
PSA	prostate-specific antigen
PTEN	phosphatase and tensin homolog
RANKL	receptor activator of nuclear factor kappa-B ligand
RICTOR	rapamycin-insensitive companion of mTOR
RNA	ribonucleic acid
RP	radical prostatectomy
rPFS	radiographic progression-free survival
RT-PCR	real time polymerase chain reaction
RT	radiotherapy
RTK	receptor tyrosine kinase
SHBG	sex hormone-binding globulin
TSC 2	tuberosis sclerosis complex 2
Vps34	vacuolar protein signal 34
